# Malaria; an Essay on the Production and Propagation of This Poison, and on the Nature and Localities of the Places by Which It Is Produced, with an Enumeration of the Diseases Caused by It, &c.

**Published:** 1830

**Authors:** 


### REVIEWS AND BIBLIOGRAPHICAL NOTICES.

---

ART. VI.—*Malaria; an Essay on the production and propogation of this poison, and on the nature and localities of the places by which it is produced: with an enumeration of the diseases caused by it, and of the means of preventing or diminishing them, both in naval and military service.*—By JOHN MACCULLOCH, M. D., F. R. S., &c. &c. Physician in ordinary to His Royal Highness Prince Leopold of Saxe Coburg. *First American Edition, Philadelphia,* 1829. 8 *vo. pp.* 219.

BY A CORRESPONDENT.

It has long been the subject of familiar observation, not only to physicians, but to the vulgar, and to the most unenlightened nations, that a production of marshes, under certain circumstances, and at peculiar seasons of the year, was the cause of intermittent and other fevers. Later and more accurate observation has shown that dysentery and cholera, with a great variety of diseases which have generally been attributed to other causes, proceed from the same source.

This mysterious poison which is so delicate as to elude the observation of our senses, and even the more accurate investigations of the chemist, is called by the British physicians, Marsh Miasma, and by the Italians, Malaria. To the greater part of the physicians and inhabitants of this country, this subject is one of great practical importance. To our merchants and travellers it is, from the extension of our commercial relations, daily acquiring additional importance. As members of the great family of mankind, we cannot be indifferent to a subject which involves so great an amount of suffering disease and death; while to men of science and literature, it will derive additional interest from the fact that this invisible agent is laying waste, and threatening entirely to depopulate some of the most interesting scenes of classic association.

Dr. Macculloch, the author of the work at the head of this article, has bestowed upon this subject a degree of research and observation that reflect upon him the highest credit, and has traced the agency of malaria in the production of disease, to cases in which its influence has been suspected by very few, even of the Medical profession.

To many of our readers his speculations will appear visionary, yet the great importance of the subject will secure his opinions a candid investigation, as the interesting and important facts which he has collected will secure to the work a very extensive circulation.

Malaria has been estimated to produce one half the mortality of the human race; and even in Great Britain, Dr. M. supposes nine-tenths of the febrile diseases which terminate fatally, are fevers of this class, which have been too often confounded with typhus.

"The value of life, of survivorship, the average chance of approaching to the proverbial limit of threescore and ten, is the measure of the salubrity of a country, and that salubrity depends mainly on the presence or absence, the range or the limitation, of Malaria. We may take the average of life among ourselves, in round numbers, at fifty, with sufficient safety for this purpose. In Holland it is twenty-five; the half of human life is cut off at one blow, and the executioner is Malaria; for there is no other cause for the superior mortality of that country. But there are districts in France where it is but twenty-two, twenty, eighteen; so little is the chance of life; while all the instruments by which Death executes his office, are here superseded by one, by that one which renders all others unnecessary, which has monopolized the functions of the whole dark catalogue—Malaria. Let us turn to Italy: the fairest portions of this fair land are a prey to this invisible enemy, its fragrant breezes are poison, the dews of its summer evenings are death. The banks of its refreshing streams, its rich and flowery meadows, the borders of its glassy lakes, the luxuriant plains of its over flowing agriculture, the valley where its aromatic shrubs regale the eye and perfume the air, these are the chosen seats of this plague, the throne of Malaria. Death here walks hand and hand with the sources of life, sparing none: the labourer reaps his harvest but to die, or he wanders amid the luxuriance of vegetation and wealth, the ghost of man, a sufferer from his cradle to his impending grave; aged even in childhood, and laying down in misery that life which was but one disease. He is even driven from some of the richest portions of this fertile yet unhappy country; and the traveller contemplates at a distance deserts, but deserts of vegetable wealth, which man dares not approach, —or he dies."

Rome is threatened with entire depopulation.

"Such also is Sicily, such Sardinia, and such is classic Greece. To live a living death, to be cut off from more than half or even that life, to be placed in the midst of wealth and enjoyment, yet not to enjoy, such is the fate of man in the lands of Europe where Malaria holds its chief seat; while in the tropical regions, it is to fall by thousands and tens of thousands, the summer harvest of death walking hand in hand with that of the vegetable world."

It is in the operations of war, however, that we are to look for the most obvious effects of this destructive agent, which has annihilated armies with the secrecy, certainty, and despatch of the Destroying Angel. Every circumstance connected with the habits, occupation and mode of life of soldiers, exposes him in a peculiar degree to the pernicious influence of malaria; and hence

"If the sword has slain its thousands, the Malaria has slain its tens of thousands. It is disease, not the field of action, which digs the grave of armies; it is Malaria by which the burning spirit, fitted for better things, is quenched, and in the coward's bed of death. This is the Destroying Angel, the real pestilence which walks at noon day; and to which all the other causes of mortality are but as feeble auxiliaries in the work of destruction."

Our Author speaks of the habit of denying the existence of danger, as a curious moral feature, characteristic of those who live in these unhealthy districts. This remark certainly does not apply to this country. The inhabitants of malarious regions being generally very willing to acknowledge the evil to its utmost extent.

### Chapter II.

*Nature of the evidences of the production of malaria.*—The power of marshes to produce remittent and intermittent fevers is generally admitted, but Dr. Macculloch intends to show that what is popularly called a marsh is not necessary for the production of malaria, and that its causes exist under numerous circumstances not at all suspected, in our own country, and at our very doors. In effecting this, we are certainly disposed to indulge him in resorting "to proofs of some delicacy," and to admit that if "exceptions do occur" they should not be allowed in so mysterious a subject to prevent a candid examination of his conclusions, when so many exceptions occur in relation to those causes of malaria, which are universally admitted!

It is a very serious error, but not a very common one in this country, to judge of the presence of malaria, solely by the occurrence of regular intermittent fever.

"The practitioners acquainted with hot countries know indeed perfectly well, that the common fever of summer and autumn, be its names what they may, is equally the produce and proof of Malaria; as this is also known to all physicians of reading, who have not had the advantage of similar experience. To them it is equally known, that dysentery is produced by the same causes, while, at present, I need not extend this enumeration.

"But this is as far from being true of the mass of domestic, untravelled, and imperfectly educated practioners, as it is of the mass of the people themselves. With these, the fevers in question are most frequently called typhus, and, further, generally considered as contagious." * * * * * "While the term bilious fever, sometimes used by others, conveys ideas no further definite, particularly as the cause is generally sought in heat, cold, fatigue, fruit even in plums and cheries specifically, and while the autumnal dysentery is attributed to the same fanciful causes.

"The real conclusion to be drawn is, that whenever remitting fevers, or fevers of whatever nature that are not contagious, as well as dysenteries (to say nothing at present of other diseases less commonly attributed to this cause,) are produced, the proof of summer Malaria is as complete as if the same soils had, in spring, produced ague; or, generally, that as the same soil, in different seasons, or under different circumstances, produces both kinds of disease, or the different species of one genus, while both are mutually interchangeable, so the occurrence of any species at any season, is a proof that the situation is productive of Malaria."

Having set aside this fallacy, he decides, "that wherever remittent fevers, or fevers of whatever kind that are not contagious, or dysenteries are produced, the proof of malaria, is as complete as if the same soil had produced ague," because the same soils, in different seasons, or under different circumstances, produce all these varieties of disease. A more delicate proof of the presence of malaria is to be found, in the fact that certain situations as compared with other situations in the same vicinity, may be said "in general terms to be unhealthy." Thus it is notorious that many families have lost, or after long suffering have recovered their health, by a simple change of residence. Dr. Macculloch very justly censures, with much severity, the loose reasoning, and phraseology of those physicians who content themselves with referring the sickness in question, to obvious physical facts, such as badness of the water, or a clay soil. A clay soil will be found to be productive of disease, only as it is favorable to standing water; and a sandy and gravel soil to be healthy, only as their loose texture admits of its ready escape.

In considering this kind of "unhealthiness," as affording evidence of the production of malaria, in places little or not at at suspected, the sickness, on rigid examination, will be found to consist in the occurence of summer and autumnal disease, or in the frequent recurrence of petty fevers, or a general febrile state that is frequently referred to dyspepsia. It will also be found, that dysentery or diarrhœa is one of the prevailing ailments, or perhaps cholera, combined with the different varieties of neuralgia.

"Even if all these should be absent, or if from fortitude, carelessness, poverty, or from weariness or contempt of physic, persons who are thus habitual and hopeless sufferers, should not give a physician all the opportunities of minute observation which he might desire, he will be a bad observer if he does not discover in the sallow complexions, the langour, the irritable tempers, or the melancholy character of individuals thus unfortunately situated, that they are suffering under fixed derangements of the larger glandular viscera; often of the liver, and perhaps much more frequently of the spleen. And should he have those opportunities of examination which severe diseases of this nature afford, he will be enabled to convince himself that this very species of disease, the noted produce of the places that notoriously generate Malaria in the hotter climates, is also habitual to the similar situations in our own country, if under a less severe character; and that it is one of the leading causes of that indefinable ill health peculiar to the situation of the patient, as it is, apparently, the great cause of so many distressing, nervous, and dyspeptic symptoms.

It is further to be remarked, that while the occasional production of intermittent fevers in these situations, demonstrates the existence of malaria, it at the same time shows, that all these diseases proceed from the same cause. Novel as these views may appear to many of our readers, they are confirmed by the fact that many analogous diseases do actually appear as the sequel of autumnal fevers, and that during the sickly season, a similar state of ill health, is common in malarious countries, even among those who escape the prevailing epidemic.

Dr. Macculloch thinks that another test of the existence of the poison, is to be found in the susceptibility of those persons who have once had remittent fever, to a new attack, on exposure to the slightest application of malaria. We agree with our author that this susceptibility sometimes continues for a considerable time—in other words it continues as long as that enlargement of the viscera, and that impaired condition of the secreting surfaces, which follow remittent fevers, continue.

"It is notorious, that those who have suffered severely and repeatedly from intermittent, or, sometimes, from remittent, become, and even through a long course of years, so highly susceptible, that the slightest exciting cause, and, among others the slightest application of Malaria, is capable of reproducing the disease. That this is true of the host of sufferers at Walcheren, needs not be said; while it has been also found common among those who have suffered from the intermittents of China or of Canada; as indeed of many other countries, among which Moldavia is said to be pre-eminent. Hence, therefore, where we find that such a person has experienced a renewal of his disorder, from communication with a place otherwise suspicious from its nature, it offers as convincing proof as can be desired, that there Malaria is produced or producible."

Such our author informs us was the susceptibility of an acquaintance, that the disease was repeatedly recalled, by merely visiting a pond dedicated to a few gold fishes. But if the slightest exciting cause will reproduce intermittents, why suspect malaria? Simple exposure to a damp atmosphere, excess in eating, or violent exercise in the sun, will do the same, during the winter, or in situations where the presence of malaria cannot be suspected. Spring intermittents we think generally arise from the predisposition acquired during the preceding autumn, and not from exposure to fresh sources of malaria—at least we do not recollect to have met with an instance of their recurrence either in spring, or the early part of summer which could not be traced to a previous attack of autumnal fever.

But as the opinions of Dr. Macculloch are entitled to the highest consideration, we will present his views, and leave others to make their own observations.

"The obscure cases in question are very principally such, that while prevention is generally easy, either by amending an existing bad situation, or avoiding an incurable one, it seems especially called for, from their number, as well as from the number and the inveteracy of the diseases thus generated. Let me urge what I said before, that Malaria produces, in itself, a far wider mass of human misery than any other cause of disease; as for the world at large, it is also the cause of far more than half the mortality of mankind. And that many of the diseases which it produces are almost beyond the power of physic, while marked by a resistance which often terminates but with life, is an additional reason for making every exertion to avoid what we know not how to cure. And further, if I am myself fully convinced by wide observation, it would also not be difficult to prove, that this very persistence, the cause of so much misery, is most generally the result, not of a fixed disease, of organic affections, as commonly supposed, or of inveterate habit, but of incautious or unknown, and repeated or continued exposure to the re-exciting cause, Malaria.

"Hence, if the diseases from this cause are incurable in the places where they arose, as is notorious in every marked situation of this nature throughout Europe, so it is from repeated or continued exposures to Malaria that they are renewed, even under change of place; and from ignorance, generally, of the situations, too often unsuspected, which are productive of poison. He who labours under an incurable intermittent in England, is perhaps sent to Italy or France for change of air and a cure; as thousands are daily sent, for other reasons, to the same countries. The physician forgets, and the patient knows not, that he is flying Scylla to rush into Charybdis, and too often confirms the disease or meets the death which he meant to avoid; while he who hopes to leave consumption behind him at Montpellier, leaves it indeed in the grave where the fever has superseded his more tardy enemy; as he who flies from poverty and England to the banks of the Rhone or the Loire, finds too late that he has bartered his health for the expected ease and happiness of France."

### Chapter III.

*On the soils and situations which most commonly produce malaria.*—Since the evil consequences of fresh water marshes are universally admitted, it is unnecessary to enter into the consideration of them. There is, however, a very popular impression, that salt water marshes are innocent in this respect. Proofs of their deleterious nature are however so abundant, throughout the whole of the Southern part of Europe, on the shores of the Atlantic, and Mediteranean, and on the Asiatic, and American continents, that it is unnecessary to particularize them. Indeed the fact has been generally admitted by those acquainted with the experiments of Sir John Pringle, shewing that small quantities of salt accelerate the progress of putrefaction; and upon this principle, it has been asserted by many of the French and Italian writers, that salt marshes are more pernicious than fresh.

The popular error, that the pernicious quality of such soils is remedied when washed by the sea, Dr. Macculloch refutes by referring to some very unhealthy situations in England, which are covered by the tide twice a day; while the palm and mangrove rivers of tropical climates, in innumerable instances, may be adduced in evidence to the same effect.

Forests in tropical climates are supposed to be fruitful sources of malaria.

"The power of woods in generating Malaria is not less notorious than that of marshes, at least in the tropical climates. To repeat all that has been written respecting this particular class of soils would be to compile without purpose. The jungles and the jungle fevers of India are as familiar; even to the multitude, as the ditches aad fevers of Walcheren. The jungle, it must however be remarked, is a low dense brushwood, or a thicket of reeds and grass; and it is often, consequently, as the residence of moisture and decaying vegetation, analagous to a marsh. Yet the production of fever does not seem limited to this particular species of woods in India; since, according to the testimony of Buchanan, confirmed by that of others in several parts of the East, fevers are produced among the opener and larger forests, in Mysore and elsewhere, and are in fact the usual concomitants of all woods.

"Yet in this matter, and even in those climates, there appears some irregularity, as far at least as we can judge from the reports of ordinary travellers; since it is said that, in Cambodia, Cochinchina, and Siam, there are extensive tracts of wood where fevers are unknown. As to Africa, the same rule seems to apply, as far as it is known to us; and the same also seems true of the warmer regions of America, however the opener and drier pine forests may be exempt."

The pine woods of our Southern states, if the test "of general ill health," laid down by our author be admitted, are very generally productive of miasmata; since sallow anasarcous countenances, and bloated abdomens are proverbial characteristics of their inhabitants, by which they may be recognised, as readily as the Esquimaux by the peculiarities of his structure.

Although woods in temperate climates are viewed with less suspicion, yet Dr. Macculloch mentions several instances not only in England, but also in Wales, where it is difficult to trace intermittent or remittent fevers to any other source, than "plashy woods and coppices, and that, even when these are situated upon the steep declivities of hills."

The experience of the inhabitants of the newly settled parts of the United States, we think, would be rather in favor of the healthfulness of woods. Newly cleared lands indeed are proverbially unhealthy, especially when surrounding hills or forests prevent free ventilation; and no doubt situations may be found in the very centre of forests, unprotected by the shade, from which exhalations will arise, the more deleterious, from the want of ventilation. But with the knowledge we already possess upon this subject, it might be supposed, independent of observation, that forests would be useful both by protecting the marshes from the rays of the sun, and from the well known influence of trees in arresting the progress of malaria, by preventing its circulation. Taking advantage of this circumstance, many of the Southern planters, by protecting the woods about their residence, enjoy an exemption from autumnal fevers, in the vicinity of very extensive marshes and mill-ponds.

Rice grounds, as the cultivation of this plant requires a succession of inundation and drainage, must be very deleterious. In support of this presumption, Dr. Macculloch refers to the great mortality which prevails among those who cultivate rice in Italy, Greece, and France, among whom, he states "on good authority," that the term of life does not exceed forty, and that the population is decimated every year. To this it may be added that the health of Savannah, since the abandonment of rice for the dry culture system, has been progressively improving, notwithstanding the abundant sources of malaria afforded by the ditches which drain these lands.

It has been said, however, that rice grounds do not produce fevers in China or India.

"In the mean time, we may at least be allowed to doubt; while if it should prove true, it is like so many other anomalies in this case, incapable of explanation for want of more accurate knowledge of the necessary facts.

"It will be difficult, however, to admit the truth of an opinion which considers the rice grounds of Bengal or similar districts as not productive of remittent fever, until it is explained whence arise those fevers which so often range in India, and of which the year 1762 produced so destructive an instance; since it was computed that, in this one season, the mortality included 30,000 natives and 800 Europeans in Bengal alone. Inundations of the Ganges may be allowed their full share, and so in many situations, may jungles; but if we exclude the rice cultivation, we shall scarcely find sufficient causes for a mortality of this nature, of which that country furnishes and has furnished at all times, abundant examples."

It might be supposed, indeed, that the extreme temperance of the inhabitants of these countries, with their great prudence in adapting their habits and modes of life to the climate in which they live, would generally protect them; so that the great mortality which occasionally prevails must be considered as fully sustaining the opinion of their insalubrity.

### Chapter IV.

*Of soils and other situations less suspected of Malaria.*—Dr. Macculloch, in speaking of the exent of soil necessasy for the production of malaria, supposes that as malaria, when wafted to the distance of several miles, and consequently when very much diluted, will produce disease, so a very small extent of marsh, if the system be brought within the reach of its influence, or "a single blade of grass acting on water may be as efficacious as an acre;" supposing of course that it is actually applied to that part of the body which can suffer from its action.

In connection with this subject, he supposes that there is so far an analogy between malaria and contagion, that with the former, as with the latter, the quality rather than the quantity of the agent, modifies the character of the disease. Thus "a minutes exposure, a single inspiration" is sometimes sufficient to produce the most fatal diseases; and in addition, we may remark that in some instances where the generation of malaria is so abundant that scarcely an individual escapes, these diseases assume the mild remittent or intermittent type; while under other circumstances, when the miasmata are so diluted that sporadic cases of fever only appear, these few cases are accompanied with symptoms of the greatest malignity. The subject must be acknowledged, however, to be attended with many inherent difficulties; and among others, so much of the character of the fever depends upon the influence of exciting causes, that it requires very minute observation, with extended opportunities, to say how much may be attributed to malaria.

Does the nature of the plants; their being ligneous or herbaceous; their chemical composition; the facility of their decomposition; or the peculiar offensive gas which escapes from some of them during this process; produce any modification of malaria? As nothing but speculation has hitherto been advanced upon this subject, which is devoid neither of interest nor practical importance, it remains to be elucidated by future observation. Supposing, however, that vegetables do not under ordinary circumstances produce malaria until their peculiar character is destroyed, we of course are disposed to decide in the negative.

As to the source of malaria.

"But to pass from this; the essential character of all marshes and swamps, as far as we can decide, is, that the land should be partially inundated, that it should be dry in some places and wet in others, or that pools and dry spots should be intermixed, or that it should be boggy and soft from the mixtures of earths and decayed vegetables with water, or that it should be subject to peculiar alternations of moisture and dryness, sometimes amounting to absolute inundation in the first case.

"Now, in all this we see no apparent reason why the water of the marsh should produce Malaria, because we do not find that water produces it in other situations; and as little have we reason to suppose it the produce of earth and water mixed, or of clay or mud; since neither is it caused by such mixture where vegetation, or vegetable matter is not present. Nor is it produced by the mixture of decomposed and subcarbonized vegetable matter and water; since it is notoriously not produced by dead peaty bogs, or by peat which carries no vegetation. The presence of vegetables or vegetable matter, therefore, in some mode or form, is necessary: while the conclusion has sometimes been, that it is a production formed between the living vegetable and water; more generally, that it is generated between that and the latter in some stage intermediate between life and absolute decomposition; or, lastly, that it is the consequence of absolute putrefaction."

The simple putrefaction of vegetables, under peculiar circumstances, will certainly produce disease of a very malignant character; but it is not so well established that it is the general, or even a frequent source of malaria. The probability is that the cause is to be looked for in what remains of the vegetable when it is so far decomposed as to form a part of the soil, constituting what is generally called vegetable mould.

Dr. Macculloch proceeds next to the examination of those situations which, not being marshes, are not generally suspected of producing malaria. The popular opinion in England, that Peat bogs are innocent is founded on imperfect observation. They do produce it in Holland and other countries; and that they do not in Great Britain is owing to accidental circumstances. The fact is that in warm climates peat is not produced, while in high latitudes malaria is seldom sufficiently active to produce disease.

There can be no doubt, Dr. Macculloch thinks, that the rushy pools and petty swamps, so common in high moorlands, produce malaria, from the fact of the occurence of intermittents in Wales, and in other situations elevated a thousand feet above the level of the sea. The minute marshy spots met with on commons and roadsides are also far from being innocent; although their limited range of action may prevent suspicion, unless when habitations are situated very near them.

"In how far meadows which cannot be called marshy are capable of producing Malaria, is an intricate and entangled question; partly on account of the difficulty of exactly limiting the term meadow, or defining the degree of moisture, and partly because they are so often intersected by drains and ditches, which may sometimes be the generative sources, instead of the including land itself. I cannot hope to clear this question by an exact definition; but taking the term in its usual lax sense, it appears unquestionable that there are many tracts of meadow, or of alluvial land, not marshy, and often not intersected by ditches, at least in a conspicuous manner, which are the sources of Malaria all over Europe."

The same fact, Dr. Macculloch thinks, occurs in innumerable instances on the continent of Europe, where no marshes are present, nothing but that drying of moist meadows, (whether previously inundated, or otherwise made wet in winter) which takes place during the summer heat.

"And it will be found, in confirmation of this, in France and in Flanders, and probably far wider than I now know, that where tracts bordering the same river, or in any other respect exactly similar, whether in soil or situation, are respectively, cultivated with grain or kept in grass, there the production of fever or of Malaria is correspondent; occupying the uncultivated lands so as to produce what is popularly called the *fevre du pays*, as if it was a necessary part of the order of things, and flying from those that have been ploughed for a grain cultivation.

"How the two facts, relatively, that is, a grain cultivation or pasture, act in this case, it ought to be almost superfluous to say; since the former husbandry will be commonly adopted whenever the meadow can be maintained in such a state of drainage as to fit it for the plough, while a condemnation to pasture is also, in itself, almost evidence of wet land. But I must here also add, what I am obliged to remark elsewhere, that the mere act of ploughing, with the crops and processes which follow it, produce a very different effect as to the natural moisture of the soil, from that which must occur under a dense covering of luxuriant grass."

Dr. Macculloch is disposed to extend the same principle, "to upland meadows, and even to perpetual pastures on the declivities of hills, to all meadow and pasture land, which is retentive of moisture, or which the winter leave a poachy and soft in the spring."

On this point, although not prepared to speak positively, we think that luxuriant vegetation acts as a preventative against malaria, and that if meadows do produce malaria, it will generally be found under circumstances similar to the following; either the vegetation has been removed by mowing or pasturage, or destroyed by the extreme heat and drought of summer, so as to leave the soil exposed to the direct rays of the sun; or that they are liable to be overflowed and covered with the soil from the adjacent high grounds, so as to expose a surface somewhat analogous to a marsh.

Dr. Macculloch, next refers to running streams and rivers. We would expect a very general admission of the salubrity of the rapid streams of mountainous countries. Yet a very common opinion to the contrary is prevalent in France; and Volney states that every river in America he visited, whether rapid or stagnant, produces malaria and fevers. It would be superfluous to say, that with regard to this country, M. Volney was much mistaken. The most rapid streams, however, may be occasionally retarded in their course, and have extensive tracts of alluvial land on their banks, and hence produce bowel complaints or other autumnal diseases, even in very elevated regions. As they approach a more level country, and especially, when subject to alterations in altitude from floods or the tide, their banks present a surface precisely analogous to a marsh, in all those circumstances which, so far as we are able to judge, produce malaria.

Dr. Macculloch is a most decided enemy to those streams, artificial lakes, canals, and ponds, which are introduced into pleasure grounds for the sake of ornament. "In one instance, the recurrence of an intermittent fever was caused repeatedly in a susceptible subject, by merely entering a garden containing a pond dedicated to gold fishes and river gods."—Such "delicate susceptibilities," if it were not for the inconvenience attending them, would be very important to our pioneer settlers, in selecting their habitations.

Canals, from their banks possessing all the properties of a marsh, and from the numerous receptacles for stagnant water, which are frequently to be found near them, will often produce malaria. In support of this opinion, are adduced the canals of France, Holland and Batavia. We may add also the experience of New York and Pennsylvania.* It is not improbable again under other circumstances, that by draining low marshy lands, they may have a beneficial influence. This subject, at this time, derives peculiar importance in the United States, from the great attention paid to Internal Improvement; and is perhaps not unworthy of being taken into the estimate, in deciding upon the expediency of different systems of improvement.

Ditches and drains, Dr. M. thinks, may be the source of the unhealthiness of certain low districts. In Walcheren, where the soil is dry and sandy, and in the Campagna di Roma, where the soil is also dry, the malaria is attributed to this cause.

Although a general opinion of the insalubrity of the drains of towns and houses prevails, yet there is a want of absolute evidence on the subject. Perhaps the history of the epidemics of our Eastern cities will afford sufficient proof of their deleterious nature. It is a question of some importance whether the fevers thus produced are typhus. The evidence adduced by both parties, (for every thing is fair in Medical Politics,) proves both sides of the question. Dr. M. offers one which he thinks decisive.

> "This is, that in the Salpetriere at Paris, intermittents were common among the residents confined there, and that the Malaria having been suspected to enter the house from the drains, the disease was, at once and forever, removed by making alterations in them."

If malaria is produced by vegetable decomposition, the general contents of these receptacles would favour the same

*It may not be improper to mention, however, that since 1823, some causes, not very well ascertained, have produced more autumnal diseases in those states, and in the middle states generally, than had previously occurred since the first settlement of the country.

opinion; and above all, this view is supported by the season of the year at which these diseases make their appearance.

In speaking of the moats and ditches, which surround fortifications, Dr. M. alludes again to the prevalent error of confounding typhus with the diseases of malaria—a mistake which leads to improper modes of treatment and prevention, besides "tending to confuse the whole history of epidemics."

"But while it is not very obvious why typhus should be generated out of the habits of a baronial castle, where every defender was almost for ever in the air, it is abundantly easy to understand how remittent and dysentery might arise from confinement within a ditch, aided perhaps by scanty food.

"And further, this view is confirmed by the analogous consequences occurring to the beseiged, and very frequently also to the beseigers, in the case of modern fortifications. There is often the same bad or scanty food, with similar fatigue and exposure in the night as well as in the day; while the works to be defended are either surrounded by water, or, if the ditch be not wet, it is seldom truly dry in those flat countries which are the most frequent seats of fortified towns. And, in fact, even in peace, these fortified places are generally unhealthy, and productive of the diseases of Malaria, as will be discovered on inquiry by any who will be at the trouble of making it; while it is scarcely less easy to ascertain, that, in most of these cases, the focus of the disease is in the ditch, and that the attack, or the fever, falls first on the sentries who mount the night guards on the ravelins or the glacis."

But why should this mistake lead to improper modes of treatment? The diseases of Malaria are indefinitely varied by the circumstances of the individual, and the nature of the exciting causes, so that emetics and bark can no more be appropriated to these than champhor and wine to typhus. Indeed it will scarcely be questioned, that under some peculiar circumstances, fevers really remittent, may become truly typhus, and (if typhus be contagious,) reproduce the same disease, or that either of these diseases may become the exciting cause of the other——in other words, that the predisposing causes of remittent and typhus fever may be so combined, that diseases will arise, having, to a certain degree, the type of the former, with the peculiar symptoms of the latter. Indeed Dr. M. makes a similar concession in a subsequent part of the work.

"The facts proving that remittents and dysenteries unquestionably arising from Malaria, have in their progress or continuance, become contagions diseases, though not so originally, are numerous, and rest on as good authorities as physic has to produce. * * * * "The assertion, or the fact, does not imply that the fever of any one individual did reproduce, through his secretions, a Malaria similar to the original one, and therefore a fever or dysentery of Malaria; but merely, that under such a state of disease, the morbid secretions in question became the source as well as the matter of the contagion; as happens even with respect to healthy persons under peculiar and well known circumstances. The original disease disappeared, and a new one was produced; or, in the morbid individual, one fever was converted into another of a different character; just as, under other diseases, a contagious hospital disorder of any nature may be superinduced on the first, even to its extinction. Or, it is not that the remittent fever of marshes is contagious, but that it may become, or give way to, a contagious fever. It is perhaps a certain heat of temper excited in maintaining a recent hypothesis, which seems to have viewed this subject in a different and unfounded light, leading to much superfluous and some angry writing."

Lakes in high latitudes, with bold rocky shores, cannot be very dangerous, and lovers of poetry and romance may be allowed to indulge their sensibilities without being alarmed by our authors fears, even if occasional reeds and water lilies should excite some suspicion. When they lie in flat level countries, their banks of course partake of all the properties of marshes.

"But it must also be said in explanation, (a view which is important, as it concerns all waters of this nature, even to pools,) that in France, it is supposed that malaria is not solely produced by the vegetating marsh, but is disengaged from the mud which the summer leaves dry, (a fact which I must notice again) and that it also escapes from the bottom and through the water, accompanying the air which is so notedlyextricated in those cases. And in confirmation of this, it said, that while such pools retain a considerable depth of water, or whenever their banks are steep, no malaria is produced, but that it appears in the reverse cases, or, either on the diminution of the water in depth, or on its retiring from the shores."

For our own part, we are content with "a sufficient reason," and have no disposition, when we have an obvious cause in the mud on the banks, to seek for imaginary ones in the vegetating margin, or in the mud at the bottom.

It is unnecessary to follow our author in his remarks on mill dams. Their insalubrity in this country depends very much upon their locality, and the nature of their banks, and still more, upon the latitude. It may not be improper to mention the popular opinion that they are less unhealthy, when supplied by a large stream; and so far as a full supply of water will keep them at a more uniform altitude, the popular opinion is not destitute of foundation.

The fact that pasture land, when broken up for the first time for cultivation, produces malaria of a virulent character, is mentioned by Volney, Rush, and Cassan, and in the West Indies has long been known as a very dangerous operation; the laborers sometimes dying on the spot, if they remain on the ground during the night.

"It would be well worth the trouble of those whose local situations give them the means, to inquire whether this, and many other analogous agricultural processes, now little suspected, are not causes of the fevers which sometimes appear in rural situations in such an inexplicable manner, when these cannot be accounted for by stagnant waters of various kinds, or by such neglected spots as I have been pointing out. The remark is of value, be the solution what it may; because the remedy will be found in breaking up such lands in June, or in May, if the summer be the necessary period, or what is preferable, in the middle of winter; since the decomposition will then take place at a time in which experience has shown that Malaria is scarcely generated in our own country, nor indeed generally in Europe. In the case of lands recently recovered by drainage, this precaution is peculiarly deserving of attention, because in this case the danger is greatest: and the same is equally true of woods, the mere felling of which sometimes disengages or produces Malaria, as is a much more certain consequence where, as in America, and as I have elsewhere noticed, these woods are broken up for cultivation."

Lands laid dry for the purpose of agriculture, or for the express purpose of destroying malaria, have frequently become much more unhealthy than before.

"It is not difficult to understand that a swamp in which the water is so deep as to impede the growth of as many plants as a drier surface would carry, will produce proportionally less of the poison in question; and that a similar diminution or under proportion of malaria will attend such a tract of land if it should contain many pools or spots divested of all vegetation. In such a case, we can conceive a certain state of drainage, such as to increase the vegetating surface, without being at the same time complete enough to check the production of Malaria; or a small quantity of poisonous marsh might thus become a large surface of wet and noxious meadow land.

"Thus also, it is not difficult to imagine how the drainage of a lake attended by a noxious margin, might increase the extent of such a description of soil, though the water itself were diminished or exterminated, and a considerable tract of dry land gained also to cultivation."

Perhaps also, there may be a certain state of moisture, much inferior to absolute wetness, which is more favorable to malaria. But we must again renew our protest against the error of considering a state of luxuriant vegetation as favorable to the production miasmata. The exposure of the mud from the removal of the water, and the aquatic plants which previously protected the soil, are sufficient to account for the fact. Hence too we can readily understand why the unhealthiness will sometimes continue but a single season; and upon the same principle, why ploughing by more effectually draining the land, changing the condition of the surface, and favoring the production of a different species of vegetation, will the more speedily correct the evil. The remedy will be the more complete if the land be broken up in the winter, or early in the spring.

Our limits will not permit us to follow the author in his history of the benefits which have resulted from agricultural improvements, and from private and public exertions directed to the correction of sources of malaria. We heartily concur with Dr. M., however, that the importance of the subject in a political point of view, independent of its connection with the health and happiness of so many of our citizens, would justify our government in devoting to it some portion of that talent and investigation, which has hitherto been wasted in political disputes, very little to the honor or permanent utility of the nation.

Dr. M. remarks, that the Delta of Egypt, both in its state of inundation and drainage, would lead us to suppose it to be as prolific a source of miasmata, as the worst situations in

South Africa; yet, unless we allow the plague to spring from this source, the few fevers and dysenteries, which prevail in that country, bear no proportion to what, *a protri*, we might suppose. Egypt, however, is inundated, not by deluges of rain as India, but by the overflowing of the Nile; consequently that part of the soil which is not inundated, is dry. In Bengal when the waters have subsided, the ground remains long in a miry state. In Egypt, on the contrary, such is the aridity of the atmosphere, and the heat of the sun, that the water has no sooner deserted the plains, than they are turned into an impenetrable crust. From these circumstances, therefore, we are prepared to expect that the extrication of miasmata would be on a very confined scale, compared with that which takes place in Bengal. Dr. M. informs us that so far as his observation extends, when intermittents occur on elevated situations, they may always be traced to marshes, on the spot, or in the vicinity.

"It is thus that I find the highest parts of Brittany to be subject to inveterate ague, even on the summits of granite hills reported to be dry land, as for example, at Carhaix. Thus also was it reported that certain hilly situations in Wales were productive of agues, and as was thought, without a cause: but the slender observation which detected that cause in this latter case, would probably have discovered it in the former, and in hundreds more, equally objects of surprise or mystery. Let the ground be carefully examined, both on the spot, and in the neighborhood, and let the possible propagation by means of the winds be also traced by the rules deducible from what I shall hereafter say on that subject, and it is probable that the far greater number of these imaginary difficulties would no longer want their solutions."

Dr. M. refers to the different opinions respecting the immediate origin of malaria; some supposing that putrefaction is necessary; others that living vegetation is an essential ingredient; others again denying that ordinary putrefaction of vegetable matter, attended with stench, can produce the poison. He thinks that this question is set at rest, by the observations of Dr. Rush, respecting the heap of coffee which produced so much excitement in Philadelphia in —93. We so far believe in *final causes*, as to suppose that our alfactory nerves were bestowed upon us for some useful purpose, and consequently are ready to believe that vegetable putrefaction may sometimes be very injurious. We cannot, however, avoid thinking that too much stress has been laid upon this said heap of coffee; else, why the simultaneous appearance of the yellow fever on the seaboard, from Boston to Savannah. The same objection will prevent Dr. M. from drawing any support from this fact, in favor of his suggestion that a difference in the nature of the plants, may produce a difference in the character of the malaria.

The process of steeping hemp and flax, and the manufacture of indigo, are spoken of as sources of malaria. We doubt whether the injurious effects of the latter occupation can be attributed to malaria in the usual acceptation of the term, as the evil appears to exist in a more tangible shape.

Dr. M. supposes that Malaria is produced at sea, by that peculiar action of water upon the casks by which carburetted hydrogen is produced; and the suspicion attaches still more strongly to bilge water; especially, when accompanied by the leakage from sugar casks, which is known to be peculiarly offensive. Numerous facts tend to prove that fevers frequently spring from these sources, especially in ships engaged in the sugar trade.

How far "mere mud, the produce of the sea, or left by the recess of the tide, can produce fevers is a question rather of curiosity than of practical importance; as the facts which might be adduced in evidence, can scarcely be separated from those in which depositions of mud are brought down by rivers, or in which marshes covered with a rich alluvial soil, exist in the vicinity. The sandy beach of our Southern states, even in Florida, is proverbial for its healthfulness. On the contrary Dr. Ferguson relates cases where, in the West Indies, the pure sand of the sea shore produces fever of the most malignant character.

The suddenness with which diseases appear on the commencement of the rainy season in Africa, led Park to suppose, that it was brought on by the mere presence of moisture; and has induced others to suppose that malaria previously generated, and diffused through the higher regions of the atmosphere, was brought down by the rain. We think the observations of Dr. M. upon this subject much more rational.

"The facts, as more accurately stated by other travellers, and also in some measure by himself in other places, seem to be, that as the vegetable decomposition necessarily active in so hot a climate, commences as soon as the ground becomes wetted, the generation of malaria begins, perhaps even on the first day or hour; and that, in this, as in every other case, it is but the produce of a vegetating soil, rendered suddenly marshy or wet under a high temperature. That this is the view entertained by the natives themselves, is plain, from the care with which they retire to their houses and endeavor to exclude even the least access of the external air: judging, what is probably true, that the whole atmosphere is one wide body of Malaria. As the rain increases, however, and the ground becomes thoroughly wetted, the diseases diminish, returning again as the retiring of the rains allows it to dry. Hence it is, that in Africa and elsewhere, the greatest influence of the Malaria takes place at the end of the rainy season; and thus also it was on the retiring of the waters after the rains and the inundation, that the great mortality commenced among our troops at Rangoon in October."

The diseases of hot climates are frequently attributed to the water. It is by no means improbable that bad water may produce bowel complaints, but as remittent fevers do not make their appearance where other causes of malaria do not exist, it at most can only act as an exciting cause.

Our author closes this chapter with an estimate of the comparative healthfulness of ancient and modern Italy; and an attempt to elucidate this difficult and interesting subject.

Rome, and the country in its vicinity, was densely inhabited at a very early period of its history. In the reign of Servius Tullus, the city contained 80,000 men capable of bearing arms. The plain of Latium, which is now a desert, was rich and populous; and in the time of the Volsci the Pontine marshes contained twenty-three towns and villages; while many places which, with the citizens of Rome, were favorite resorts for health and amusement, are now uninhabitable. Indeed the whole tenor of the history of the republic proves that many districts which are now desolated by malaria, were inhabited by powerful and warlike nations, that contended obstinately with Rome for the dominion of Italy.

From the want of medical writers, we can form no idea of the extent to which autumnal diseases prevailed in the vicinity of Rome, during the period of the republic; since history, even in modern times, never condescends to more than a passing notice of epidemics, unless they are so serious as to interfere with the military operations of governments. The country, however, abounded in the sources of miasmata; and we are furnished with sufficient evidence of its suffering severely from them. Not to dwell upon other facts, the first settlers were compelled to abandon the Aventine mount by the pernicious exhalations from the neighboring marshes; and in addition to this, the city itself was repeatedly ravaged by pestilential diseases, which in all probability arose from malaria.

"It is further probable that the larger proportion of pestilences described by the Roman writers, were unusually severe visitations of the marsh fever; though at this distance of time, and under information which is not medical, we must not absolutely decide that some of these may not have been instances of contagious fever, perhaps even of plague. Such probably may have been the pestilences of 355 and 573, since there are facts in Livy's narrative which rather seem to justify this conclusion; and such possibly were the cases in which the city alone suffered, while the surrounding country was exempt. But allowing even much for this, we find from Plutarch, that noted periods of sickness occurred in the time of Romulus and in that of Numa, while similar ones are recorded of the reigns of Servius Tullus and Tarquinius Superbus: and when Livy says that in the short period of 173 years, or, from 287 U. C. to 460, there occurred at Rome or in the surrounding country, no less than nineteen distinct plagues, none of them at longer intervals than seventeen years, and some lasting two or three years together, it is not possible to avoid concluding that the fever of Malaria must have prevailed then in as great severity as it does at present."

It is difficult, however, to make the comparison. The great wealth of ancient Rome would have supported a very dense population under very disadvantageous circumstances. On the other hand, Italy, at present, is suffering from all those causes of moral and political degradation which must attend the decay of feudal institutions that have been raised by fortuitous circumstances above their natural condition in society—a nobility proud, poor, degenerate and licentious—a people corrupt and oppressed, without industry or enterprise—the landed estate fallen into the possession of a few speculators, who, intent upon present gain, are too illiberal in their views to form any extended plan of public improvement.

Malaria is generally found to diminish with the progress of agricultural improvement, and independent of the great extent to which this would be carried, in a country so rich and populous as ancient Italy, the cultivation of the soil was considered as an honorable and dignified employment, by the most illustrious men; whose intelligence and unbounded wealth enabled them to execute every plan that might conduce to the ornament or salubrity of the country. The laboring classes were temperate, and protected by laborious habits, and uniform exposure, against the usual exciting causes of disease.

The depressed condition of agriculture in modern Italy has condemned the unhealthy districts to pasturage. The laborers, who are occasionally employed to collect the harvest, reside at a distance, are ill clothed, and ill fed. In summer there prevails in these vallies a suffocating heat during the day, which is succeeded in the evening, from the change of the wind to the north, by an intolerable cold. It is not, therefore, a matter of surprise if the laborers, when exhausted by fatigue and the heat of the day, exposed, as they are, to the cold night air, almost without clothing or shelter, should contract fatal diseases. These considerations are confirmed by the fact that these diseases are always intermittent; since it cannot have escaped the observation of any person familiar with the diseases of warm climates, how much the application of cold, under circumstances of this kind, disposes them to assume the form of a fatal typhoid intermittent.

Other causes perhaps may be found in the gradual change of climate, which has been taking place in Europe for centuries, and which, perhaps, has virtually placed Italy ten or fifteen degrees further to the south. Something perhaps may be attributed to the destruction of the groves which were interspersed throughout Italy, which, as some suppose, were associated with the religious rites of that country, with a view to this very object. An additional cause may be sought for in the geological changes which are constantly taking place.

"Every river which, by bringing down materials from the mountains and propelling the sea, has formed flat tracts of land, such as the great plain of Bengal, or those of the Oroonoko, and the Mississippi, with thousands more of less note and on all scales of dimension, must sometimes rise above its ordinary banks, causing inundations. Thus, when incommoding the adjoining lands in agricultural countries, it is embanked by art; while, in consequence of this process, the alluvial matters which would be dispersed over large spaces, from those changes of direction whence the plains themselves are formed, become confined in the channel itself: depositing within a narrow space on its bottom. Thus they tend to raise the water in its bed, and, consequently, to cause it, on any increase, to overflow, still more certainly, the lands around. And as this effect is the very consequence of the embankment, so, at any given point, the bank must be made to keep pace with the rise of the channel, that the restraint may be effectual and constant. Hence as the river becomes more elevated, the ultimate result is the same as if the surrounding lands had been depressed to the same amount: and thus, while the stream which drained them once can drain them no longer, they become, first, meadows, and ultimately marshes. And if, in the former condition, they can still be drained by means of canals and floodgates, this process becomes in time inefficient, and recourse must be had, as in Holland, to lifting machinery."

"It is thus, in the district in question, that the Po is now, over a large tract, literally running on the summit of a wall, threatening even other dangers than a further increase of bad health which it is producing: both circumstances united having led to a design of letting it loose to find a new channel for itself, which, had the government of Italy continued in the same powerful hands long enough, would doubtless have been carried into execution."

From these political and natural causes, the climate of Italy is becoming very unhealthy. Her population is declining, because the tribute of the world is no longer paid to her political power, or her religious influence; and the pestilence, as the inhabitants retire before it, extends its ravages and multiplies its strength.

In the mean time, the interest of her natural scenery appears to increase with the progress of her desolation. Nature, as if in mockery of man, appears in her most bewitching attire, and holds out to him, in the richest profusion, treasures of luxuriant vegetation, which he cannot enjoy. Her fragrant atmosphere is loaded with the invisible messengers of death, and her brilliant sun and beautiful sky serve but to light the passage to the grave.

The evil is no doubt, to a great degree, within the control of industry and art; and an intelligent and laborious peasantry, favored with freedom, might arrest the evil, and confine it within very narrow limits; but if the present system of civil, political, and religious oppression is continued, the "Eternal City," will soon exist only in the pages of history.

### Chapter VI.

*On revolutions and changes which take place with regard to the production of Malaria, whether from natural causes or from artificial sanitary measures.*—The subjects of this chapter have been in a great measure anticipated in the preceding pages.

A progressive amelioration in climate has generally resulted from agricultural improvement. The most prominent cause of amelioration is the drainage of swamps and marshes; but as the malarious surface is thus sometimes increased, or a state of moisture produced more favorable to the generation of miasmata, the evil is frequently aggravated. Under these circumstances the remedy, for reasons already assigned, will be more complete, if the soil be occasionally subjected to the plough. It follows from these premises, that as the population of a country becomes dense, and the proportion of arable land is increased, it will become more healthy; and on the contrary, when it is in a declining condition, the sources of malaria will extend, and disease prevail in an accelerated ratio.

The removal of forests may very materially affect the health of the surrounding country. Much of the sickness of newly settled countries, may be attributed to the exposure of lands which have been kept in a marshy condition from the want of cultivation, or to the removal of so many obstacles to the diffusion of malaria; and in climates favorable to the production of miasmata, the evil will probably be increased as the clearing advances, until the density of the population brings every portion of the arable land into requisition. It has been already mentioned that the unhealthiness of Rome has been attributed to the destruction of the forests which protected it from the unhealthy exhalations of the surrounding country.

Forests, on the other hand, may increase the virulence of malaria by preventing their diffusion; and in such cases, an opening sufficient to admit of a free circulation of air, becomes a matter of some consequence.

It may sometimes become a question, whether the evils arising from a marsh may not be remedied, by completely laying it under water; but the experiment certainly is one, not to be adopted without great circumspection. In connection with this, it may be mentioned that in very wet, and consequently very sickly seasons, the most unhealthy situations are frequently remarkable for their exemption from disease.

It is unnecessary to mention the effects which may be produced by changes in the courses of rivers, eruptions of the sea and the possible draining of the Northern Lakes, &c. as the effects will correspond with the principles already advanced.

We have already, in speaking of the malaria of Italy, alluded to the effect of alluvial deposites at the mouths of rivers. We shall, in connection with this subject, take the liberty of making an extract from a succeeding chapter.

"The former great cause, unceasing, if slow, is that indeed in which the whole world is implicated; nor is it difficult to see, as I formerly explained, that while the mountains shall flow to the sea, increasing the plains, and, in creating new lands, providing, in an increase of fertility as well as of extent, for an augmenting population, so must the augmentation of insalubrity and the increase of diseases accompany these changes. And while, in such cases, increase of industry and attention is required to meet the evil, it is the misfortune of Greece, as it has been of many other declining or fallen states, to have suffered a loss or diminution of both, or of that at least which alone could have maintained and stimulated them, from the effects of its unhappy and lamentable political condition. Did any visionary geologist thus choose to speculate on the day when the mountains shall be levelled with the plains beneath, an event which, in a period beyond the range of calculation, must happen should our globe endure thus long, he must also be prepared to view that previous period when, whatever vegetation or whatever animals may possess the marshes and plains of that world, it will scarcely be man; since, long before this, he will have been driven from them, as he has from the Pontine marshes, by that plague which nature is even now daily preparing in silence to keep his unmanageable increase within bounds."

It is gratifying to know that the prospect of these evils is so very remote, that we need entertain very little uneasiness on the subject, for long before this day arrives we shall be quietly asleep in the lap of mother earth, and little of our blood will run in the veins of any living creature.

### Chapter VII.

*On the propagation of malaria.*—As malaria has hitherto eluded our researches, we can at present only consider it as a component part of the atmosphere, and its diffusion must consequently be regulated by those laws which govern atmospheric currents. Its union with the air, whether it be a simple mixture, or chemical combination, is probably influenced by the temperature, electricity and moisture of the latter, and perhaps by the degree of intimacy with which the aqueous particles are held in combination. To what extent malaria is modified by these changing conditions, has hitherto been the subject only of fruitless speculation, and vague analogies—so many difficulties interpose to prevent accurate and satisfactory observation. We only know that in some conditions of the atmosphere, malaria is much more active than in others, whether it be that is taken up under some circumstances more readily, or that under others, its properties are destroyed, by too close a combination with the air, we pretend not to decide; and only allude to the subject, because the unauthorised confidence of some authors appears to render it necessary.

The most obvious circumstance which occurs to us in considering the effects produced by malaria, is proximity of situation; and it is both reasonable and consistent with experience, to estimate the danger by the distance, as well as by the extent of the pernicious soil. Many instances, however, may be mentioned in which this circumstance has been strangely overlooked, in the selection of the sites of the towns, both in ancient and modern times; and in none more so than in European settlements in the tropical climates. To the history of modern war, however, we are to look for the most serious and lamentable, and we may add, the most inexcusable errors of this kind. The thousands of lives, which are thus uselessly and often recklessly sacrificed, should certainly recommend this subject to the consideration of government, and induce them to make it a part of the studies of a military education. We would be pleased to introduce some of our author's observations on this subject, but it is needless to dwell upon particular instances when such numerous facts are before the world.

The second circumstance mentioned by Dr. M. is "condensation." It is easy to suppose that while the generation of malaria continues, if not dissipated or destroyed, it must accumulate. Thus experience proves that malaria, when confined within the walls of a forest, as frequently occurs in newly settled countries, and the marshy ground of jungles, or within valleys where the surrounding hills do not admit of free ventilation, will acquire unusual virulence. It is frequently remarked in the southern part of Italy, that the wider the mouth of the valley towards the sea, and the less deeply it penetrates among the hills, it is the more unhealthy.

It is a very curious and important fact, which the present degree of our information does not permit us to explain, that persons living at a distance from the marsh sometimes suffer severely, while those in the immediate vicinity enjoy a comparative, perhaps an entire exemption. It is frequently the case that ranges of hills are particularly affected, while the intermediate low grounds escape. "If this is not to be explained," says Dr. M. "by the flow of a current of air, so directed as to escape the low grounds and declivities, while it ranges along the hills in contact, I have no solution to offer."

It is generally the case that the situations nearest the surface of the earth suffer most, and it is said that the distance between the different stories of a house frequently makes a very important difference. The reverse of this, however, frequently occurs, and when malaria impinges on the acclivity of a hill it is frequently raised to a very considerable elevation.

"But I will add one statement, extracted from Captain Smyth's valuable statistical table of Sicily, because it appears to generalize the whole of these facts; leading to the conclusion, that, in nearly an equal number of cases, the higher grounds suffer as much as the lower; the locally healthy as those which are the very seats of Malaria. In this document out of seventy-six unhealthy towns and villages enumerated, there are thirty-five situated on hills or declivities; while, from personal information, I may add that many of them are at considerable distances from the tracts which produce the disease. And I may add one more remark as to the theory of this propagation, derived from a writer on the climate of Italy. It is, that the southern winds in that country, propagate along the hills, upwards, that Malaria which the northern or mountain ones do not; such winds, independently of their superior power in producing the pernicious exhalations, tending, from their temperature, to ascend the acclivities, while the colder winds, as is easily understood, have the opposite inclination."

Are these facts connected with the specific gravity of malaria? or of that portion of the air with which it most readily combines?

It may easily be conceived that vallies not only condense, but may direct the course of malaria, since winds, when their course at all corresponds with the direction of a valley, frequently sweep along it.

We have already remarked the influence of trees upon malaria; we will again take the liberty of referring to the subject for the purpose of quoting some interesting remarks of the author.

"Lancisi remarks that in later times, there was extirpated near Rome, a forest to the southward, reaching from the heights of Frascati and Albano to the Tiber, and protecting it from the malaria so abundantly generated in that quarter by these marshes. Thus, says he, was destruction first let in upon the Campagna: but it is since that date that a similar proceeding seems to have opened Rome itself in another quarter to the Malaria of this immediate tract of pernicious land.

"If my information at least is correct, there was formerly, at this point, or in a situation interposed between the Campagna and Porta del Popolo, a wood, cutting off the communication through the northeast winds; and it is since the destruction of this, that the new progress of this pest, so remarkable, has been noticed.

"The evil, spreading as it were, from a fixed point, and making, in every year, a further step, so as gradually to drive the inhabitants before it, as far at least as these are opulent, and able to quit their unhealthy habitations; since not only does poverty check the migration of the lower classes, but, from the causes hereafter to be stated, their crowded streets are far less affected by this poison than the dwellings of the rich.

According to these reports, it appears to enter at the Porta del Popolo, and from the north-eastward; while it may be suspected here, that as far as this occurrence is new, as it is asserted to be, the immediate cause must be sought in the extirpation of the mass of wood just mentioned, which formerly sheltered this quarter of the city from that wind which crossed the pestiferous plain."

Some facts appear to prove that malaria may be retained in situations where it has not been generated.

"To name a striking case of this nature, I may refer to Valetta, alluded to on a former occasion, on account of the consequences which almost invariably occurred in the Floriana guard, while other parts were little affected, or retained their health. Here, the ditch was very deep and narrow, and so perfectly dry that it could not be suspected of producing the malaria to which the effects in question were owing. Nor could this be explained, except by supposing that it lodged and protected from dissipation, a current of noxious air, produced from that salt marsh which seems to be the source of Malaria in Valetta, and which the sea breeze directed on to this spot."

It would also, like contagion, appear to have the power of attaching to certain substances; although it cannot, like it, be transferred from place to place.

"In the Campagna of Rome, it is remarked that if the laborers cut down certain plants, (a bushy thistle, chiefly, of which the botanical character has escaped me,) a fever, that would otherwise not have occurred, is the consequence. The Malaria seems, or is thought, to be entangled within it and to be let loose by this disturbance."

We make another extract, for the purpose of pointing out an instance of very hasty inference in our author.

"Further, it is a common remark in many parts of Italy, that as long as the labourers are in the erect posture, they incur little danger, but that the fever attacks those who sit down or lie on the ground, as if the poisonous matter extended to but a small altitude above it. Whether, in this case, the cause be, as in the former, an attachment of the Malaria to the ground itself, or to its vegetation, or whether it is that it is a part of a ponderous stratum of air, lying on the ground, as carbonic acid does in the the Solfatara, is a question which requires investigation, at least as far as philosophy is concerned."

The general statement is, that during the day, the labourers may lie down with impunity, and the increased danger unquestionably arises from lying down, and perhaps falling asleep, on the damp earth in the cold evening air.

Does a damp atmosphere favor the production or transmission of malaria, or merely act as an exciting cause of disease? Some analogies, drawn from the ready diffusion of the matter of contagion in this condition of the air, appear to favor the former of these opinions. Malaria too appears to be dissipated or destroyed by the heat of the sun, and rendered active by the evening dew.

"Thus also is it especially remarked, that if a hot day is succeeded by a cold and damp night, the effects of Malaria are much augmented; and the same analogy holds as to similar changes in seasons, or as to incidental ones occurring in any manner. Hence if cold and wet weather should unexpectedly take place in the midst of a hot summer, an augmentation of severity, or a state of disease before not in existence, will occur; and hence also severe epidemics occur particularly, if, to such a hot summer there should succeed a cold and rainy autumn; the production of the poison, as I formerly remarked, being apparently augmented in this manner, while the atmosphere is also rendered a better conductor. The general philosophy applicable to all these cases, is, that watery vapour, or a moist atmosphere, is the best solvent and conductor of Malaria, as a dry one is the worst; while independently of the different effects of those two states on vegetation and on putrefaction, it is the effect of the sun to evaporate or disperse, possibly in a great measure to decompose, that gaseous matter, which is condensed, and probably very often again precipitated, during the evening, together with the vapors which had been held in solution in the air."

If the fact, however, be true that malaria cannot be transmitted over a considerable body of water, it would appear that water has the power of destroying its activity, and by parity of reasoning, we might conclude that a very damp state of the weather was unfavorable to its production. That fogs are not peculiarly adapted to holding it in solution is further proved by the fact, that malaria is arrested by any intervening obstacle, as trees, houses, &c., while the fog spreads over the whole surrounding country. We have uniformly observed that very rainy weather, in sickly seasons, never fails to arrest the progress of disease, and if followed by cool northernly winds, to check it permanently. Cold autumns are unhealthy, only when cold nights are alternated with days sufficiently warm to generate malaria, and give the former full activity as an exciting cause of disease.

A damp atmosphere may act by producing a degree of moisture favorable to the generation of malaria, and the warm southerly winds, which frequently accompany this condition of the weather, must conduce to the same effect.—Under other circumstances, it will operate by its chilliness as an exciting cause; and when we take this into consideration with the fact, that the seeds of autumnal fever frequently lie a long time dormant in the system, before they are rendered active by an exciting cause, we may justly conclude that the opinions of our author are to be received with great reservation.

It is unnecessary to repeat the evidence advanced by numerous authors to prove, that independent of any peculiar condition of the human body, malaria is infinitely more destructive by night, or rather, that it is in the night only that any serious danger is to be apprehended. In Italy an opinion is prevalent that it is the evening air only that is pernicious, and that after nine or ten o'clock, the extrication, or, as it is here supposed to be, the deposition of malaria ceases.

The night air and the evening dews of summer are a source of alarm in all climates. Dr. M. thinks, and the opinion is no doubt just of many places where, from the absence of intermittents, the cause is never suspected, that the danger really arises from malaria; since the coldest nights of winter seldom produce disease. Yet we may be permitted to add, that Dr. M. in his zealous search after malaria, has overlooked the numerous reasons why night air should be more injurious, in summer, than in winter.

On the subject of *prevention*, Dr. M. is disposed to attribute more efficacy, than we think they deserve, to the use of narcoties—as opium, tobacco, &c. On the subject of wine and generous living, he inclines to the opinions of the old school that they are useful. Luxurious habits will generally warp the opinions of those who are disposed to indulge their appetites, but the united testimony of American physicians, and of European surgeons who have served in the East and West Indies; the universal practice of the aborigines of tropical climates; and the comparative exemption which the French and Spaniards enjoy in these climates from their temperate mode of living; establish the fact that a diet, principally of vegetable food, and as sparing as is compatible with the necessary labours of the individual, furnishes the best protection.

On the influence of fires, Dr. M. remarks.

"That in this case they may act, at least in two ways, is obvious; that is, by drying the surrounding air and thus diminishing its conducting power, and by producing a dispersive ventilation, or bringing a salubrious mass of air on an unhealty point. As to facts in proof of the utility of fires, Lancisi points it out as to Rome; and even Pliny, long ago, declares the same opinion, quoting, further, the authorities of Empedocles and Hippocrates to the same effect. That Napoleon took the same view of their use, adopting this expedient very largely, and with success, when his armies were occupied in the very worst district of Italy, is a specimen of military experience which may save the necessity of quoting others less decisive. One very pointed case, of a civil nature, is also worth recording. In this case, the superintendant engaged in directing the cutting of wood in Africa, erected thirty earthen furnaces on the spot where his men were employed, lighting them every day. Before this, he had always from forty to forty-eight of his workmen sick; when in a short time, they were reduced to twelve, then to four, and finally to one."

The cold air of the night probably, besides acting as an exciting cause of disease, renders the body more susceptible to the impression of malaria. Hence the importance of guarding against it by kindling fires, and carefully accommodating the clothing to atmospheric vicissitudes—a practice that cannot be too closely followed by all who are much exposed in unhealthy climates.

It is a fact so familiar to the aboriginal inhabitants of all tropical climates, as to influence their habits, that excesses in eating, during the heat of the day, are more injurious than at any other time. Inattention to the obvious dictates of nature, indicated by the general languor and loss of appetite which are commonly felt at this time, is probably the exciting cause of a very large proportion of the cases of bilious fever of hot climates.

Intermittents spread with less facility in populous towns; and it is particularly worthy of remark as forming a very decided contrast to what we observe of contagion, that the closest and most filthy streets are the most effectually protected. Dr. M. attributes this to the decomposition of the malaria by fires and smoke. But why go in search of hypothesis, when we have a mechanical obstruction amply sufficient to account for the fact, supported by the analogous influence of trees and rising grounds in obviating the deleterious effects of miasmata?

Great diversity of opinion exists as to the distance to which malaria may be wafted by winds. On a subject which is certainly within the reach of observation, arguments drawn from meteorological phenomena are certainly entitled to very little weight. The difficulty consists in distinguishing the effect produced by a distant marsh, from that of more obscure sources of malaria in the immediate vicinity. We have good authority, however, founded on accurate observation, for supposing that, by a steady uniform wind, it may be carried to the distance of three or four miles, and even much further.

A very obvious mode of determining the question might be supposed to be found in ships lying at anchor in the neighborhood of marshes, were it not for the fact that the diffusion of malaria is arrested, by any considerable body of water. Rush states that ships lying in the middle of the Delaware at Philadelphia escaped the fatal epidemic of 1793. Dr. Blane also states that the crews of the ships which attended the fatal Walcheren expedition, and lay within two or three hundred yards from the shore, were not affected by the exhalations from the land. Other facts equally conclusive in their character are related by the same author. Some facts, however, are related by Dr. Johnson, which apparently tend to prove that miasmata, which have ascended during the day into the higher regions of the atmosphere, may be brought down with the evening dews, and thus produce an impression farther from the shore, than they could be directly wafted by the wind.

"Having occasion," says he, "to take a passage from Madras to Calcutta in a foreign merchantman, at that time, I sat late on deck, one evening after our arrival in the Ganges, the vessel being at anchor a mile from the shore, and not a breath of wind moving in any direction.

As the dews began to fall, I perceived, all at once, a faint heavy odour, to account for which I was much puzzled, as there was no breeze to waft any exhalation from the adjacent shores. My reflections were soon interrupted, however, by a sense of faintness, giddiness and at length nausea, with which I was suddenly affected. I immediately went below, not a little alarmed, and fully persuaded that I was seized with the fever, whose effects I had so much reason to dread.

On drinking some warm water, to clear my stomach, I took a dose of calomel and opium, and next morning castor oil. Although no further symptoms of fever occurred, yet I felt an unusual degree of lassitude and depression of spirits for some days after I got to Calcutta."

This fact cannot be considered as invalidating the conclusion that miasmata are absorbed or rendered inactive by passing over water, for the impression left upon the mind of Dr. Johnson was, that they could not have come immediately from the shore.

Dr. M. however is disposed to give it a much more extensive range.

"That within moderate limits, such as four, five, six, or more miles, the fever, or the malaria, is blown off the shore to the ships, I have found distinctly recorded in ships' journals in different instances; as I have also been repeatedly informed that the smell becomes immediately sensible; while officers aware of this danger on these coasts, quit the deck and go below, at the moment they perceive this change to have taken place, or even, on some occasions weigh their anchors and run to sea, And though I have quoted one specific case of this nature in the Medical part of this work, the accuracy of proof which it affords is so remarkable, that I must notice it here. In this instance, with a healthy ship and crew, anchored at least four miles from the shore, a sudden change of wind brought out the smell of the land, on which orders were immediately given for weighing; while, even before the cable chain could be cleared, most of the men working at it, who were the only ones first permitted on deck, were seized with disease which proved the fatal cholera to the greater number."

When we think, however, of deciding the question from isolated facts of this kind, it is constantly to be borne in mind, that ships frequently carry with them local causes of malaria, and that the bodies of the men from previous exposure may be already predisposed to disease.

Dr. M. however, is not even contented with these more extended limits, but supposes that the spring intermittents, popularly attributed to the East wind, do in reality arise from Malaria transported from Holland. We are very willing to admit with our author that an East wind, simply as an East wind, can do no harm—that it is a bad wind when it blows under evil circumstances—that it may be hot and dry in one place, bitter cold in another, and damp and foggy in a third—also that in Flanders as on the Eastern coast of the United States,

"It is the south and the south west-winds that propagate the diseases of Malaria; and the reason will be evident on inspecting the geography of this tract. And there, it is the north, and also the north east, which remove them, because they blow from the sea; though charged with fogs to darken the whole land."

But Dr. M. proceeds to say, that in Scotland, where intermittents from a local cause are unknown, they are produced by the East wind, and that clear and unaccompanied with fogs.

"Thus, for example, on a sudden change of the wind to the east, soldiers in the Castle of Edinburgh, susceptible of the disease from former habits, become immediately affected, when a north or west wind, equally cold, does not produce the same effects: while, in other places equally free of the endemic disease, a new disorder is even produced in a new subject."

And lest he should not find intermittents enough to serve his purpose, he intends to prove in a future volume, that those indefined and undefinable sufferings, usually attributed to nervous and hypochondriacal affections which have been equally the "jest of poetry and prose"—the jest of rude health, ignorance and selfishness, do in reality arise from malaria brought from Holland.

We object to our author's eloquence as ill timed, and to his reasoning as not to the purpose. The East wind has not the power of originally producing fevers unless it conveys malaria, but it has the power of reviving them by the cold damp fogs which accompany it. He acknowledges a predisposition from former disease, and this predisposition we fancy is sufficient for the explanation, without supposing a new application of malaria. We have ourselves experienced the renewal of intermittents, with all those terrible feelings which our author compassionates so sincerely, and which we attributed, not to the malaria accompanying the East wind, for it was at a season of the year when malaria had not been called out by the genial heat of the summer sun, nor to the cold accompanying this wind, because the colder north winds have rather the opposite effect, but simply to their dampness acting upon predispositions acquired during the preceding autumn.

### Chapter VIII.

*On the influence of climate and season in modifying malaria.*— Is the production of malaria modified by changes of season and temperature, or is its influence in producing disease thus modified? Dr. M. carries the former of these opinions, so far, as to suppose that climate, temperature, &c. have no material influence in modifying the type or character of the fever, but that the varieties are produced by a difference in the evolution of malaria—a length, we apprehend, to which very few of his readers will follow him. On the contrary, we believe that all diseases produced by malaria are essentially intermittent, and that the variations from this type are produced by the influence of constitutional or exciting causes.

We have generally been disposed to attribute spring intermittents to the predisposition acquired on the preceding autumn. Dr. M. attributes them to malaria generated at the time. In temperate climates they seldom attack those who have not had fever on the preceding autumn. They are more numerous after a mild winter; Dr. M. accounts for this last fact by supposing an incomplete destruction of vegetation, and a degree of temperature favorable to the generation of miasmata. We have rather been disposed to attribute it to the effect produced upon the individual. We see an effect of cold weather analogous, although leading to a different result, in the fact that persons, who have had the yellow fever in one of the southern cities, lose the immunity they derive from that circumstance, by spending a winter in a northern climate. Sufficient heat and moisture being all that is necessary to produce malaria, a very dry, or a very wet season, under different circumstances of soil and location may be equally productive of disease.

Through the geography of malaria, which forms the subject of the next chapter, our limits do not permit us to follow our author very extensively. For one remark we were unprepared.

"But there is a general accusation against the whole of the shores of the Baltic; against Denmark very widely, and especially also against Samogitia and Courland: while Stockholm is familiarly known for the severity and inveteracy of its intermittents: so little is a northern latitude a security, where there are wet lands and a hot summer. The marshy forests of the Kama and the Viatka in Poland, are equally notorious; and it would appear indeed that the fevers of Malaria abound in greater severity, generally, through all the woody and marshy regions of these countries, even when placed far to the north; since even Lapland itself does not appear to be exempt."

Few Americans we think will read the following without a smile.

"I might detail the geography of the malaria of America at considerably more length, since the information here is more perfect, but that I am unwilling to extend a chapter, the length of which is already formidable, and since it is, practically, less interesting than the countries especially frequented by our travellers, to which I have, preferably, given the room which I cold spare. Generally therefore, I must observe, this plague occupies the Canadas, in summer, every where along the shores of the great lakes and rivers; the character being commonly that of inveterate intermittents; while, whether Lake Erie is worse than the others or not, it has the worst reputation, as have, elsewhere, the banks of the Mohawk river, and the Genesee. With respect to the United States, formed of alluvial land as they chiefly are, Volney remarks, that out of a space of three hundred leagues, he did not find twenty houses free from the fevers of Malaria: the most distinguished places, nevertheless, being the course of the Ohio, rendering even the interior state of Kentucky insalubrious, the towns of Norfolk, Charleston, New York, Philadelphia, New Orleans, Savannah, and Pensacola, with Baltimore, and almost the entire states, in fact, of Virginia, the Carolinas, Georgia, the Floridas, and Louisiana.

"Such are the United Provinces of this great country; while in many of the Southern States, the evil has, for some time, been so rapidly increasing, as almost to threaten the abandonment of the lands; the proprietors having in reality, in many parts, been obliged to quit their houses and estates and retire to the towns, even against an express law which compels their actual residence; thus leaving them to the superintendance and occupation of their slaves, and offering a strong argument in favor of a black population at least, if not of an enslaved one. But to cut short this portion, the same plague is found to prevail very widely even through the extreme interior, and amid the newly settled or still uncleared lands, along the course of the Mississippi and its endless tributary streams; to the no small surprise and the serious grievance of the numerous settlers by which such a state of things was not contemplated. What the fate of much of this new country may ultimately be in this respect, it is difficult to foresee, when we reflect on the numerous circumstances already stated, which modify the production and propagation of Malaria, and where so much is yet to be done as to alteration; though it is to be suspected that no changes and no cultivation will ever bring into a state of salubrity, a country so abounding with alluvial plains, even in the interior, and so extensively the produce of its numerous and enormous rivers."

Notwithstanding our authors gloomy predictions, we incur no risk in asserting that, with the exception of the alluvial soils on the large southern water courses, there is scarcely a situation in which an individual, by avoiding unnecessary exposure to exciting causes, and by exercising a prudent caution in his diet, may not escape autumnal disease.

We pass over the chapter on the nature of malaria as the various hypothesis on this subject, have not even the shadow of probabilty to recommend them, and our author has no new theory to advance.

### Chapter XI.

*On the general effects of malaria upon the constitution and on the diseases which seem to be produced by it.—*

"That the residence of successive generations in a district of this nature produces a degeneracy of the races, is amply shown in various parts of France and Italy; and never more distinctly than when the inhabitants of the marshy plains and vallies come into immediate contact with a people of the same radical origin and race, inhabiting the healthy, mountainous or hilly tracts which bound or include these. The stature not only becomes reduced, but deformities are frequent; while anatomically the bones are found to be affected; their extremities in particular being unusually large and spongy, and rickets, as a positive disease, being also an implicated consequence.

"The color of the skin, and the general superficial aspect of the people in these cases, has never failed to attract the attention of even the most cursory travellers. The former is sallow, or yellow, or else stained with different hues, and in extreme cases, has even a livid appearance: while, to a medical examination, it is found to pit on pressure; this condition often amounting to absolute œdema, and the muscles being soft, yielding, and unelastic. Such persons have often the appearance of being fat; but this, when it exists, is wanting in firmness, as if a great part of the accumulation consisted of water in the cellular membrane. It is also to be remarked that the hair is flaccid and the beard scanty; while, in the most poisonous regions of France, it is further asserted that pale hair abounds, when in more healthy places, the very same race is noted for the darker tints. A dull languid eye, very often also yellow, is a circumstance which has attracted general attention.

"An enlargement of the abdomen, commencing sometimes even from birth, and often rendered the more conspicuous from the slenderness and emaciation of the limbs, is also a feature which no traveller has overlooked; and it is often in itself sufficient to demonstrate the nature of the place where these wretched beings are doomed to live, or rather, as the inhabitants of the Pontine marshes express it, to die. That the very form and extent of the liver can often be traced externally, by the eye, is an anatomical fact, belonging to this state of things: while an investigation after death discovers various diseased structures in that organ, in the spleen, and in the mesenteric glands; together with water in the cellular membrane, and a general enlargement of the whole lymphatic system. In the Pontine marshes, the residents have the appearance of walking spectres; being often also œdematous all over, and thus dragging on a miserable existence through the short term of their wretched lives. That the inhabitants of such districts have a late puberty and are less prolific than in healthier regions, is a fact which has been asserted and again contradicted; yet it is one which could not excite surprise should it be proved.

"There is nothing in these pernicious countries more striking to a cursory traveller, than the appearance of age which occurs at a very early period of life. Even the children are frequently wrinkled; and, in France, in perhaps all the worst districts, a young woman, almost even before twenty, has the aspect of fifty; while, in men, the age of forty is equivalent to sixty in healthier countries, both in appearance aud vigor; the very few who live to fifty, appearing to have arrived at the protracted term of fourscore. Of personal beauty in females, there appears to be little trace at any time; but whatever may have existed is rarely prolonged beyond seventeen. And the expression keeps pace with all else; being that of unhappiness, stupidity, and apathy: an habitual melancholy which nothing can rouse, and an insensibility to almost every thing which operates on the feelings of mankind in general. A slow and languid speech, a similar languor in the walk and in all the actions, indicate equally the condition of the mind and of the body in these wretched countries.

"That the period between thirty-five and fifty is the most hazardous and diseased portion of this diseased and miserable life, is a very general remark in all the regions subject to malaria; while it is not less generally observed, that those who survive this period, often live to become old; frequently also recovering a certain po: tion of health which might have been lost. Of another general effect which has been asserted to exist, it seems reasonable to entertain some doubts; since it is an assured fact, that a high degree of nervous irritability, both mental and bodily, is a frequent attendant upon the chronic condition of the fevers of malaria. The assertion is, that the people in question are, generally, little irritable, or even sensible; and sometimes, to such a degree, as scarcely to express the feelings of pain, even under surgical operations.

"That apathy which was just noticed as expressed in the physiognomy, is a character which influences the whole conduct of these degraded and unfortunate beings. Seeking solitude, shunning society and amusements alike, without affections, without interest in any thing, they make no exertions to better their condition; not even to avoid the sources of danger that surround them, or to take the most common precautions that are pointed out: while, attached to the soil, from habit or indolence rather than from regard, they will not be convinced of its nature or dangers; fatalists in practice and even in belief, and refusing to admit that there is any other lot in life than that which is their own.

"That such a condition is a frequent result of marsh fevers, and very particularly under improper treatment, is a fact which I must notice in the medical part of this work: but even independently of this, such debility of the intellect seems to be the produce of the insensible action of this poison on the nervous system: a circumstance that indeed might naturally be expected from physiological considerations connected with the general influence which malaria exerts on the body. And that this condition is even propagated, seems, further, fully proved; so that an universal degeneracy of mind and body both, appears to be the certain lot of those races which a combination of unfortunate circumstances have placed in countries that seem to have been intended, rather for the habitations of reptiles and insects than for those of man."

Our author thinks it probable that the stupidity of the ancient Bœotians, and of the modern Hollanders, arises from malaria. It is to be regretted that this influence was not known to Buffon, as it might, in the absence of better arguments, have very materially assisted him in establishing his favorite theory of the degeneracy of the human race in America.

After alluding to fevers, our author speaks of dysentery, cholera and diarrhea as diseases of a marshy country. That these diseases are, for the most part produced by malaria, will be generally admitted. They require, however, a peculiar modification of the exciting causes of disease to call them into action, and with respect to the two latter, it may be remarked that they are occasioned by miasmata, of a degree of concentration much less than is necessary to produce fever, and that frequently they arise from the injurious effects of great heat and sudden changes of temperature upon the viscera.

In addition our author enumerates apoplexy, palsy, and visceral obstructions. To these he is disposed to add the mesenteric affection, worms, ulcers, rickets, scrofula, phthisis, scurvy, chlorosis, very probably goitre, and above all, neuralgia including sciatica, tooth-ache, and head-ache.

"I shall only add, that if the consideration of the neglected varieties of intermittent was a main inducement to the production of this work, it was the study of Neuralgia which originally led to the whole inquiry; to that primary course of observation and reflection, of which the remaining results will be submitted to the medical reader in the subsequent volumes."

No schoolboy's imagination indeed could be more active in conjuring spectres, than is our author in hunting sources of malaria; yet upon this subject, we may be permitted perhaps to remark, not only that these diseases may frequently arise from malaria, but that they will most frequently be met with in those situations, when its operation is so feeble as not to produce regular intermittents.

The old opinion of the healthful tendency of intermittents is probably not entirely destitute of foundation. Since we may observe that in those situations where annual epidemics prevail, the inhabitants are remarkable for their freedom from disease during the remainder of the year.

Having already exceeded our limits, we are compelled to take leave of the author by cordially recommending his book to the perusal of our readers. This work is chiefly valuable for the collection and arrangement of a great body of valuable facts, comprehending perhaps every thing that is certainly known upon the subject. These facts, however, have been collected from books, and hence his reasonings and his conclusions, are frequently more indefinite and inaccurate, than could readily have occurred to a person familiar with the subject from personal observation. We regret to say, that the style of the author is by no means equal to the industry and judgment displayed in collecting his materials; it is laboured, and occasionally eloquent, without attaining that perspicuity and conciseness, which are the chief merits of a work written expressly for scientific or professional readers.

